# Imidazolium-Based Ionic Liquid Exhibiting Dual Hydrophilic and Oleophobic Properties without Polar End Groups

**DOI:** 10.1021/acs.langmuir.4c04319

**Published:** 2025-01-14

**Authors:** Alan Tirado, Lei Li

**Affiliations:** Department of Chemical & Petroleum Engineering, University of Pittsburgh, Pittsburgh, Pennsylvania 15261, United States

## Abstract

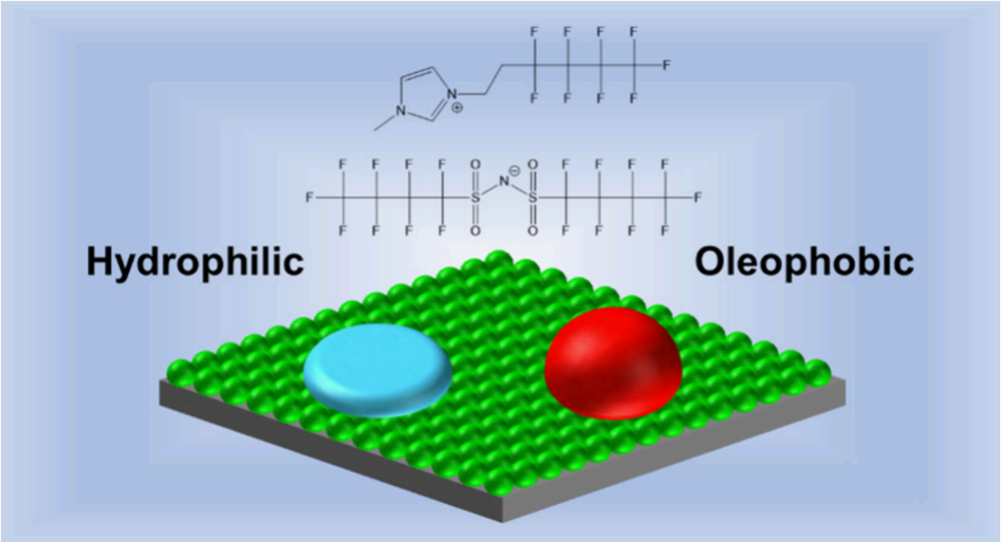

Simultaneously hydrophilic and oleophobic surfaces offer substantial advantages for applications such as antifogging, self-cleaning, and oil–water separation. It remains challenging to engineer such surfaces without requiring polar functional groups. This study introduces HFIL, a novel ionic liquid (IL) coating that achieves simultaneous hydrophilic and oleophobic properties via a one-step dip-coating process without relying on polar functional groups. Key findings show that, despite the bulk form of HFIL having a high hexadecane contact angle (HCA) of 74.1° and an even higher water contact angle (WCA) of 87.6°, the IL forms a stable monolayer on high-energy surfaces exhibiting a much lower WCA of approximately 40° with minimal change to the HCA. Washing tests demonstrate that, even without the polar functional groups, there is a non-zero bonded thickness upon which the oleophobicity is comparable to polytetrafluorethylene (PTFE). These properties highlight HFIL’s potential for durable applications in antifouling, antifogging, and environmental separation technologies, where selective liquid interactions are essential. This work contributes to a broader understanding of IL-based surface modifications, advancing the development of high-performance coatings.

## Introduction

The study of ionic liquids (ILs) has expanded significantly over the past 2 decades, establishing ILs as versatile components in advanced material applications. ILs, characterized by their unique properties, like low volatility, high thermal stability, and tunable characteristics, have proven valuable for applications in fields ranging from energy storage to surface engineering.^1,2^ One of the most compelling areas of IL research involves their use in surface wettability modification, engineering surfaces to selectively interact with different liquids, which has far-reaching implications in areas such as medical instruments, energy conservation, and environmental protection.^3^

In particular, simultaneously hydrophilic and oleophobic surfaces offer substantial advantages for applications such as long-term antifogging, detergentless cleaning, and oil–water separation.^3−5^ Traditional surface treatments achieve either hydrophilicity or oleophobicity, yet it remains challenging to engineer surfaces that combine both properties without requiring polar functional groups or complex coating procedures.^3,6^ Previously, Li et al. explored the unusual wetting behavior of nanometer-thick perfluoropolyether (PFPE) films, finding that they could achieve higher hexadecane contact angles (HCAs) than water contact angles (WCAs) on silicon substrates.^7^ This unusual behavior, in which water readily wets the surface while oil is repelled, was attributed to a kinetic limitation of the polymer coating. The study suggested that small structural “voids” within the film, formed by molecular packing or dynamic rearrangements, permit water molecules to quickly penetrate the layer and interact with the substrate, whereas larger hexadecane molecules cannot penetrate easily and, thus, interact primarily with the coating surface. These findings underscore the importance of fluorination and molecular packing in achieving simultaneous hydrophilic–oleophobic properties.

With further expansion on this concept, Wang et al. later investigated the role of end groups in PFPE coatings and their influence on dual wetting characteristics.^6^ By variation of the number of polar hydroxyl end groups, they demonstrated that only specific amounts of polar functionality could establish the appropriate interchain distances to allow water to penetrate while hindering oil. When there were no polar groups, the polymer chains remained loosely packed and mobile, allowing both water and oil to readily pass through. When there were too many polar groups, the polymer chains bonded to the surface with more frequency, creating interchain gaps that were too tight to even allow water through. The study emphasized that the unique combination of oleophobicity and hydrophilicity is achieved not only through fluorination but also by carefully controlling the end-group interactions with the substrate to ensure a stable, selective layer.^6^

In this study, we explore a novel IL, HFIL, which exhibits dual hydrophilic and oleophobic properties without the presence of polar functional groups, a feature that challenges the previous understanding established with PFPEs. This work seeks to contribute to the growing body of knowledge on ILs by examining HFIL’s unique wettability behavior and its potential implications for practical applications in surface engineering and coating technologies.

## Results and Discussion

**Figure 1:**
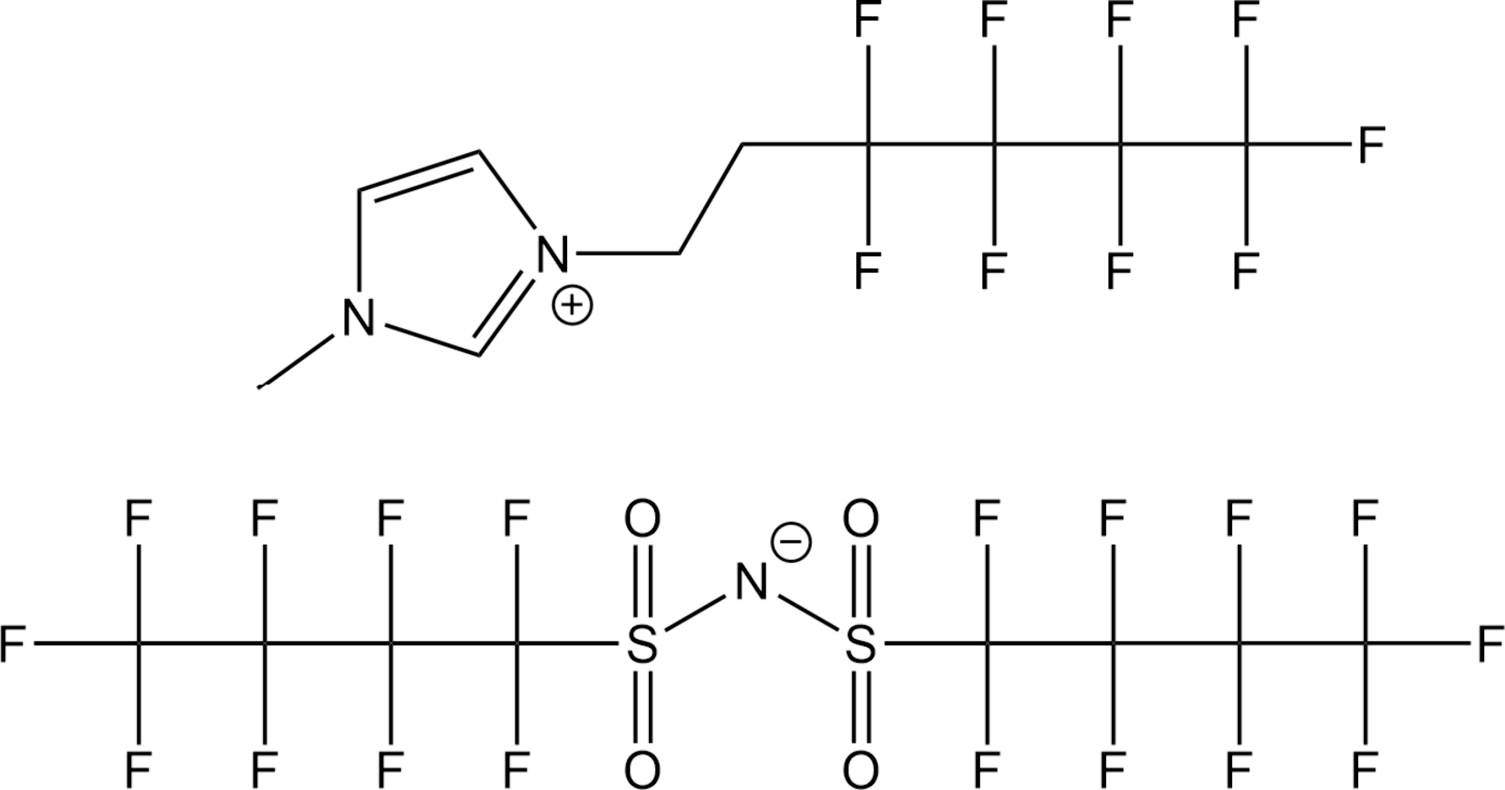
Chemical structure of HFIL.

Figure 1 shows the chemical structure of HFIL or 1–1*H*,1*H*,2*H*,2*H*-perfluorohexyl-3-methylimidazolium bis(nonafluorobutanesulfonyl)imide. At room temperature, bulk HFIL takes on a solid, largely crystalline structure with a melting point of around 85 °C.

**Figure 2:**
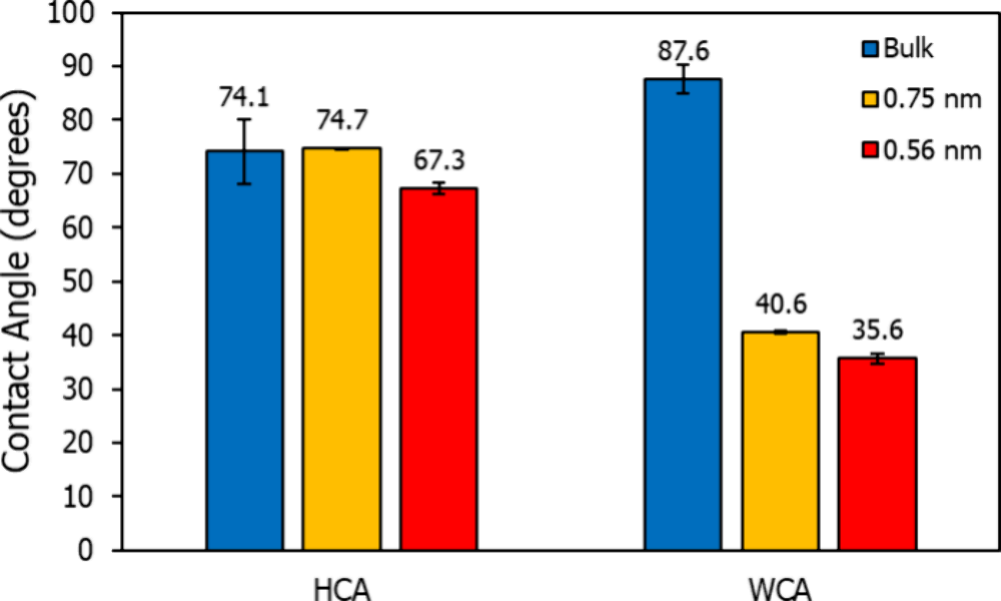
HCA and WCA of HFIL as a bulk material and as a nanometer-thick coating on a Si wafer.

Figure 2 presents the wettability of HFIL for water and hexadecane in both the bulk and thin-film states. As a bulk solid, HFIL exhibits a notably high HCA of 74.1 ± 6°, a value significantly greater than polytetrafluoroethylene (PTFE) surfaces, which typically yield HCAs of around 40–50°.^8−11^ This unusually high oleophobicity suggests that HFIL’s molecular structure repels oil more effectively than conventional oleophobic surfaces.

With the transition to the thin-film regime, specifically to a 0.75 ± 0.02 nm layer on native oxide silica, HFIL demonstrates a marked reduction in the WCA relative to the bulk IL, dropping from approximately 90° to 40°, while the HCA remains stable across both regimes. This observation aligns with the size-exclusion-based penetration mechanism for coatings with simultaneous hydrophilic–oleophobic properties. Small structural “voids” within the coating, formed by molecular packing or dynamic rearrangements, permit small water molecules to quickly penetrate the layer and interact with the substrate, whereas larger hexadecane molecules cannot penetrate easily and, thus, interact primarily with the coating surface. The surface of bare native oxide silica is both highly hydrophilic and highly oleophilic, with very low contact angles (<10°) for water and hexadecane.^12^ As the HFIL coating thickness decreases, its surface coverage diminishes, rendering the water (but not hexadecane) penetration easier and consequently reducing WCA (but not HCA) significantly, as illustrated in Figure 2.

**Figure 3:**
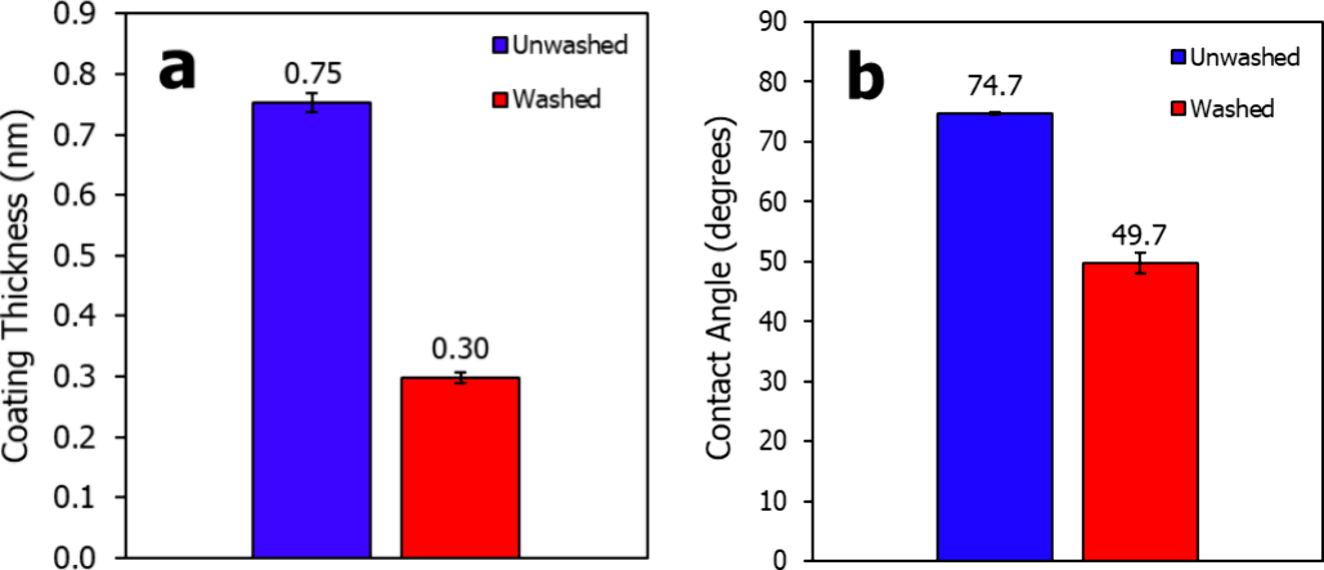
(a) Thickness of HFIL on Si wafer before and after washing and (b) resulting HCA at each state.

Prior studies indicate that polar functional groups are critical in achieving hydrophilic–oleophobic properties, as they enable coatings to “bond” with polar substrates, stabilizing the coating through specific packing arrangements. Coatings lacking these polar groups typically remain mobile on the substrate, allowing both water and hexadecane to penetrate with minimal resistance.^6,12^ However, as shown in Figure 3a, HFIL achieves a stable, sub-monolayer thickness of 0.30 ± 0.01 nm on native oxide silicon, even without polar functional groups. Additionally, the washed surface maintains a HCA of 49.7 ± 1.7°, as seen in Figure 3b, comparable to PTFE, indicating significant oleophobicity. Given this information and the fact that bulk HFIL is a solid and highly crystalline structure at room temperature, it is likely that the confined nanofilm on the substrate forms a fairly tight-packed solid-like arrangement, contributing to its stability and resistance to washing.

**Figure 4:**
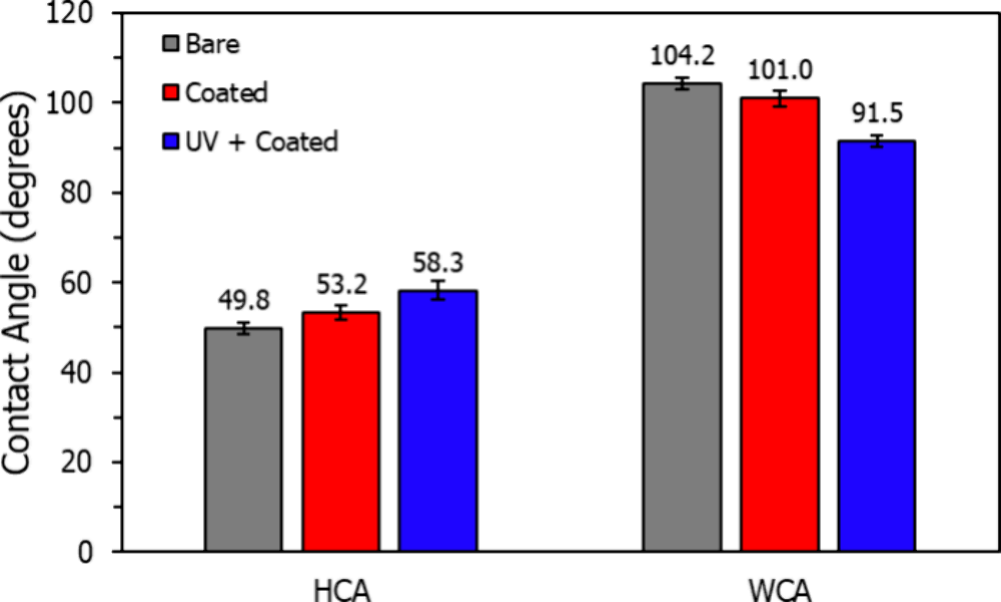
HCA and WCA of Teflon with and without HFIL coating.

Figure 4 illustrates a notably different interaction when the HFIL is applied to a PTFE substrate. Bare PTFE exhibits a HCA of 49.8 ± 1.3° and a WCA of 104.2 ± 1.3°, values consistent with the literature.^8−11,13−16^ Following immersion in HFIL solution, the HCA rises slightly to 53.2 ± 1.6°, while the WCA falls slightly to 101.0 ± 1.7°. This simultaneous increase of HCA and decrease of WCA represent the added interaction between the test liquids and the IL coating, shifting the contact angles toward those of the bulk HFIL. However, both contact angles showed minimal change, indicating that both hexadecane and water primarily interact with the PTFE surface rather than the IL coating. This outcome suggests a low bonded ratio of HFIL on PTFE, likely due to PTFE’s low surface energy, and further implies that the bonded HFIL observed on silica is not solely a result of confinement-induced ordering.^8,16^ Instead, a stronger interaction between HFIL and high-energy surfaces promotes a densely packed nanofilm that restricts hexadecane penetration.

In prior work, the surface energy of polymer substrates, such as poly(methyl methacrylate) (PMMA), polystyrene (PS), and polycarbonate (PC), has been increased using ultraviolet (UV)–ozone treatment to enhance bonding of functionalized coatings.^15,17^ Here, a clean PTFE substrate was subjected to UV–ozone treatment for 1 h before HFIL coating. Although slight shifts in contact angles were observed, the changes were minimal, suggesting a limited impact of UV–ozone treatment on HFIL bonding. These slight angle adjustments may instead reflect surface roughness changes induced by UV–ozone treatment, as previously documented.^15^

The HCA and WCA were used to calculate the surface free energy of the bulk HFIL as well as the HFIL-coated Si wafer based on the Fowkes model shown in eq 1
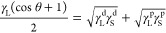
1where γ_L_ is the surface tension of the liquid, γ_L_^d^ and γ_L_^p^ are the dispersive (nonpolar) and polar components of the liquid surface tensions, respectively, and γ_S_^d^ and γ_S_^p^ are the dispersive and polar components of the surface free energy, respectively.^18^ The model assumes that the total surface energy is equal to the sum of the dispersive and polar components. Hexadecane is considered to be completely nonpolar; as such, the dispersive component is the only component of the surface tension, reported as 27.66 mN/m.^19^ For water, the polar and nonpolar components for water are reported as 43.7 and 29.1 mN/m, respectively.^20^ The results for the surface energy of HFIL and the calculated polar and nonpolar components are listed in Table 1. The bulk HFIL has a low surface energy of 20.2 mN/m, just slightly higher than that reported for PTFE.^11^ The surface energies of the thin-film coatings increase significantly relative to the bulk, reaching 59.7 mN/m on the 0.75 nm coated Si wafer and 62.4 mN/m on the 0.56 nm coated wafer.

**Table 1:** Surface Free Energy of HFIL as a Bulk Material and as Nanometer-Thick Coatings on Silicon Wafer

	γ_S_^d^ (mN/m)	γ_S_^p^ (mN/m)	γ_S_^total^ (mN/m)
bulk HFIL	11.2	9.0	20.2
0.75 nm HFIL/Si wafer	11.0	48.7	59.7
0.56 nm HFIL/Si wafer	13.3	49.1	62.4

## Conclusion

A novel imidazolium-based ionic liquid (HFIL) with no polar functional groups has been developed, which exhibits unique wettability properties. Although the bulk material is both hydrophobic and oleophobic, HFIL forms a simultaneously hydrophilic and highly oleophobic surface when applied as a nanometer-thick coating on high-energy substrates, such as silicon wafers. This behavior contrasts with previous findings that polar functional groups are essential for bonding on polar substrates; HFIL demonstrates a stable, bonded thin film that achieves oleophobicity comparable to PTFE even after washing. Attempts to coat HFIL onto PTFE revealed that this bonding effect is not solely due to confinement but rather involves specific interactions between HFIL and polar substrates. Further investigation into substrate–IL interactions may enable the design of durable IL coatings tailored to enhance surface properties in applications, such as wetting control, antifouling, and lubrication.

## Experimental Section

### Preparation of Samples

Synthesis of HFIL is described previously.^21^ The IL nanofilms are deposited onto the substrate surface via dip-coating procedures previously established in our lab. The HFIL is dissolved in Vertrel XF to prepare dilute solutions. For Si wafers, the substrates undergo 30 min of UV/ozone treatment using a BioForce Nanosciences UV/ozone procleaner [power specifications: 110 V alternating current (AC), 50/60 Hz, 0.5 A, and single phase (1 PH)] with 185 and 254 nm wavelengths in ambient air at room temperature. For PTFE substrates, the surface is washed with water followed by isopropyl alcohol and then allowed to dry at 40 °C for 10 min. The cleaned substrate is vertically submerged into and subsequently pulled out from the dilute HFIL solutions at a speed of 1 mm/s using a KSV Instrument dipcoater. The thickness of the lubricant films is directly controlled by changing the solution concentration and keeping the dip speed constant.

Washed substrates are prepared in the same fashion as described above but are subsequently dipped 3 times in pure Vertrel XF at a speed of 1 mm/s using the dipcoater to remove any mobile HFIL from the surface.

The thicknesses of the fabricated IL nanofilms on Si wafers are measured using a J.A. Woollam alpha-SE spectroscopic ellipsometer at an incident angle of 75° and a beam diameter of ∼2 mm. Optical constants of the native oxide layer are determined using the “NTVE_JAW” database complex refractive index after UV/ozone treatment but before dip coating. After dip coating, the Cauchy dispersion model is used to measure the thickness of the IL films. The measured thicknesses are the average thicknesses within the beam spot. The reported thickness is the average measured thickness across at least five points on the coated substrate.

For measurements on bulk HFIL, a mold was created using a ^3^/_8_ in. USS flat washer (0.1 in. thickness) glued to a glass slide, then washed with water followed by isopropyl alcohol, and allowed to dry at 40 °C for 10 min. Solid HFIL was added to the inner spacing of the washer and placed into an oven at 90 °C for 10 min until HFIL was fully melted. The mold was then removed from the oven and allowed to cool at room temperature for 72 h before use. A glass Petri dish covered the HFIL in the mold while it was cooled to minimize ambient hydrocarbon contamination.

### Contact Angles

The HCA and WCA of HFIL on the substrates are measured using a VCA Optima contact angle system. Testing liquid drops (0.5 μL in volume) are automatically dispensed on the surfaces, and the drop shapes are captured using a charge-coupled device (CCD) camera and analyzed using vendor-supplied software. The reported contact angles on coated surfaces are averages from individual drops deposited on at least three separate substrates with similar film thicknesses (within 0.1 nm). For uncoated surfaces (i.e., bare PTFE and bulk HFIL), the reported contact angles are the averages from at least three drops deposited on the fresh surface.
